# The Horizon of Thyroid Imaging Reporting and Data System in the Diagnostic Performance of Thyroid Nodules: Clinical Application and Future Perspectives

**DOI:** 10.17925/EE.2024.20.2.11

**Published:** 2024-07-12

**Authors:** Seyedeh Niloofar Sharafi, Mohammad Moarefzadeh, Milad Tari Moradi

**Affiliations:** 1. Nottingham University Hospital NHS Trust, Nottingham, UK; 2. Lorestan University of Medical Sciences, Lorestan, Iran; 3. Qazvin University of Medical Sciences, Qazvin, Iran

**Keywords:** Classification, diagnostic imaging, risk assessment, thyroid cancer, thyroid nodule, ultrasonography

## Abstract

The widespread occurrence of thyroid nodules and the typically slow progression of thyroid cancer have led to the development of the thyroid imaging reporting and data system (TI-RADS). The primary objectives behind the development of TI-RADS were to minimize unnecessary biopsies of non-cancerous nodules, enhance the overall precision of diagnosis and establish a uniform risk-stratification framework based on the lexicon to notify healthcare professionals of nodules that require a biopsy. The identification and precise diagnosis of thyroid nodules have led to improved clinical practice examination reports within the general population. TI-RADS is a risk-stratification system related to thyroid lesions and based on ultrasound characteristics and is similar to the structure of the breast imaging reporting and data system. There are various versions of TI-RADS, with some being widely used and adequately validated, while others lacking thorough evaluation. TI-RADS uses a numerical scoring system for characteristics, and its categories are determined by the cumulative score of a thyroid nodule, indicating the likelihood of it being benign or malignant. In this article, the various TI-RADS systems were examined as a successful method for producing precise and comprehensive documentation, with a particular emphasis on their functionality, similarities, distinctions and potential future developments.

Thyroid nodules are common worldwide, and their prevalence is increasing. Most nodules are asymptomatic and detected incidentally on cross-sectional imaging or physical examination. In rare cases (10–15%), nodules are malignant and require diagnostic evaluation. Even malignant nodules frequently show non-aggressive behaviour.^1^ The increase in the incidence of thyroid cancer is unfairly distributed globally, and the morbidity increases moderately from year to year, mainly in female patients.^2^ Moreover, the prevalence of thyroid nodules increases with increasing age. Thus, around half of women older than 70 years have a nodule.^3^ For the assessment of thyroid nodules, a diagnostic ultrasound (US) imaging, which is a widely accepted method, is suggested as the first-l ine modality.^4,5^ With the improvements in imaging technologies and increased use of diagnostic imaging, there is a considerable increase in the rates of nodule detection, discrimination between malignant and benign nodules and fine-needle aspiration (FNA), which are necessary for clinical decisions.^6,7^

The increase in the diagnosis, despite its limited impact on survival rates, primarily stems from the relatively indolent progression of the most common form of thyroid cancer, known as papillary thyroid cancer (PTC).^8^ PTCs frequently manifest without any noticeable symptoms. Autopsy findings reveal that approximately 11% of individuals possess one or more foci of PTC in either the thyroid gland or a nearby lymph node.^9–11^ The rapid increase in the occurrence rates of thyroid cancer has been attributed to the identification of this asymptomatic condition.^12^ Moreover, colloid goitres, which are a prevalent, non-cancerous growth in the thyroid gland, can present as either a diffuse or a nodular pattern. It is crucial to distinguish them from other potential forms of goitre, particularly malignancy. A comprehensive understanding of the condition is necessary for an accurate diagnosis. The initial evaluation heavily relies on patient history and physical examination, with a focus on identifying the characteristics indicative of malignancy. Thyroid US and serum thyrotropin levels are the primary techniques used to assess colloid goitres and rule out other thyroid abnormalities.^13^

Studies have demonstrated that the widespread accessibility and usage of ultrasonography for detecting nodules and assisting in needle biopsy procedures have been linked to the increasing rates of detection.^14^

Therefore, improving diagnostic techniques and using advanced technologies are essential for safer thyroid surgery, reducing the uncertainties and risks associated with thyroid nodules. Surgical removal of the nodule becomes a viable option when biopsy results are inconclusive.^15^ To address the high number of potentially unnoticed PTCs and the associated concerns of excessive treatment and rising costs, the American Association of Clinical Endocrinologists (AACE) and the American Thyroid Association (ATA) have issued recommendations for a more cautious approach.^16,17^ The goal is to stabilize the rates of occurrence of thyroid cancer and decrease overdiagnosis and overtreatment.^17–19^ As a result, a reasonable, reliable and more cost-effective system for risk stratification can be established.

Innovations in molecular testing and novel therapies have altered the approach to aggressive thyroid cancer, impacting management recommendations for early-stage and progressive diseases.^20^ Various classification systems and methodologies have been published to categorize thyroid nodules based on their risk of cancer.^21,22^ To improve the effectiveness of US diagnosis, Horvath et al. set up the first thyroid imaging reporting and data system (TI-RADS) based on the US features of thyroid nodules in 2009, and since then, there have been various versions of TI-RADS across the world.^23^ Multiple factors contribute to the existence of numerous TI-RADSs, each with unique advantages. TI-RADS aids physicians in precise diagnoses, which is modelled based on the breast imaging reporting and data system (BI-RADS). TI-RADS uses a standardized scoring system to categorize thyroid nodules based on their risk levels. This system has proven to be highly accurate in diagnosing thyroid nodules through US, resulting in a reduction in biopsies of benign nodules. However, it is important to acknowledge that TI-RADS also has its limitations. These include the potential for similarities and discrepancies in terminology and standards when different researchers establish their own classification systems for describing and defining the US features of thyroid nodules.^24^

The TI-RADS serves multiple purposes, resulting in a lack of consensus and ongoing debate regarding its validation.^25^ Various categorization schemes exist for different versions of TI-RADS, allowing users to determine when to use the FNA method, conduct US follow-ups on suspicious nodules and safely disregard benign or non-suspicious nodules.^26^ The scoring system consists of five classifications based on US findings. As the cumulative score increases, the corresponding TI-RADS level also increases, indicating a higher probability of malignancy.^27^ The present study evaluates different TI-RADS techniques as diagnostic methods, focusing on their functionality, similarities, differences, challenges and potential future outcomes.

## Literature search strategy

We conducted a comprehensive literature review by exploring PubMed, Scopus and Google Scholar databases for English-l anguage articles published prior to 30 December 2023. Our search criteria encompassed various terms, such as ‘thyroid cancer’, ‘thyroid nodules’, ‘thyroid imaging’, ‘TI-RADS’, ‘diagnosis’ and ‘risk-stratification’. The abstracts of the identified articles were assessed for their relevance, and subsequently, the complete texts of all pertinent publications were obtained. Furthermore, we meticulously examined the references cited within the selected articles.

## Different risk classification systems for thyroid nodules

Various risk classification systems have been released for thyroid nodules, specifically emphasizing US characterization. These systems differ in their methodology, with some using simple pattern recognition, while others using intricate patterns, weighted risk and multiple risk categories. They integrate a blend of nodule morphology and size metrics.

### The American Thyroid Association system

The ATA system, an evidence-based recommendation and an atlas of sonographic features, serves as a valuable resource for healthcare practitioners in the management of thyroid nodules and thyroid cancer.^28^ It was published in 2006 and updated in 2009 and 2015.^29^ The ATA system introduces a novel classification that encompasses five distinct categories: (1) benign (risk of malignancy: <1%), (2) extremely low suspicion (risk of malignancy: <3% in lesions measuring 20 mm or larger), (3) low suspicion (risk of malignancy: 5–10% in lesions measuring 15 mm or larger), (4) intermediate suspicion (risk of malignancy: 10–20% in lesions measuring 10 mm or larger) and (5) high suspicion (risk of malignancy 70–90% in lesions measuring 10 mm or larger).^30^

### Kwak thyroid imaging reporting and data system

In Kwak TI-RADS, a total score for each thyroid nodule is based on five sonographic nodule features: (1) solid component, (2) hypoechogenicity or marked hypoechogenicity, (3) micro-l obulated or irregular margin, (4) microcalcification and (5) taller-than-wide shape, and it is calculated to define the need for fine-needle biopsy. This simple scoring system has more function in the classification of thyroid nodules in a clinical setting. This reporting system was modified in 2011 and 2013.^21,31^ The Kwak TI-RADS classification system consists of five distinct categories: TI-RADS 1 indicates a normal thyroid; TI-RADS 2 signifies the absence of any suspicious features and suggests a benign condition; TI-RADS 3 indicates the absence of any suspicious features but carries a high probability of being a benign nodule; TI-RADS 4a denotes the presence of one suspicious feature; TI-RADS 4b signifies the presence of two suspicious features; TI-RADS 4c indicates the presence of three or four suspicious features and, finally, TI-RADS 5 suggests the presence of five suspicious features.^32^

### Korean Society of Thyroid Radiology thyroid imaging reporting and data system

The Korean Society of Thyroid Radiology TI-RADS (K-TIRADS) was developed in 2011 and revised in 2016. K-TIRADS uses sonographic characteristics to determine the necessity of a biopsy based on risk classification into four distinct categories. The risk of the malignancy is assessed by three suspicious US features in K-TIRADS, which include a shape that is taller-than-wide and a margin that is spiculated or micro-lobulated, presence of microcalcification and other sonographic features related to the composition and echogenicity of the nodule (*Table 1*).^33^

### European Thyroid Association thyroid imaging reporting and data system

European TI-RADS (EU-TIRADS) was described by the European Thyroid Association for the US assessment of thyroid nodules in 2017.^34^ This model aims to diagnose malignancy with high sensitivity and to maintain high negative predictive value. It was modelled based on the BI-R ADS in 2009.^23,34^ This reporting system was altered and validated based on a prospective study. This is a five-stage system with the ability to describe, recognize pattern findings on US and measure size.^35^ Details of EU-TIRADS and the risk of malignancy are shown in*Table 2*.^34^

### American College of Radiology thyroid imaging reporting and data system

The American College of Radiology TI-RADS (ACR TI-RADS) was introduced in 2017 and uses a standardized scoring system for reports.^26^ This system comprises five categories ranging from benign to extremely suspicious nodules. It provides specific recommendations for FNA and sonographic follow-up based on a combination of category and nodule size.^36^

**Table 1:** Korean Society of Thyroid Radiology thyroid imaging reporting and data system categories^33^

K-TIRADS categories	Definition	US features	Risk of malignancy (%)
1	No nodule	NA	<1
2	The nodule is benign	Spongiform Partial cystic composition with comet-tail artefact Pure cyst	<3
3	Low suspicion of malignancy	Partially cystic composition without any US feature Solid isoechoic/hyperechoic composition without any US feature	3–15
4	Intermediate suspicion of malignancy	Solid hypoechoic composition without any of the three US features Partial cystic composition with any of the three US features Solid iso/hyperechoic composition with any of the three US features	15–50
5	High suspicion of malignancy	Solid hypoechoic composition with any of the three US features	>60

*K-TIRADS = Korean Society of Thyroid Radiology thyroid imaging reporting and data system; NA = not applicable; US = ultrasound.*

The primary objective of the American College of Radiology (ACR) Committee in developing TI-RADS was to create a set of guidelines for managing thyroid nodules that are incidentally detected on positron emission tomography (PET), computed tomography (CT), magnetic resonance imaging (MRI) or US.^37^ Additionally, they aimed to devise a comprehensive lexicon that could effectively describe all thyroid nodules observed through sonography.^38^ All thyroid nodules can be categorized with TI-RADS classifications based on the lexicon published by the ACR.^27^ This category was presented by Horvath et al., based on the US-assessed thyroid nodules and cumulative score.^36^*Table 3* outlines five categories: TI-RADS 1 (TR1), benign; TR2, not suspicious; TR3, mildly suspicious; TR4, moderately suspicious and TR5, highly suspicious for malignancy.^26,39^

**Figure 1:**
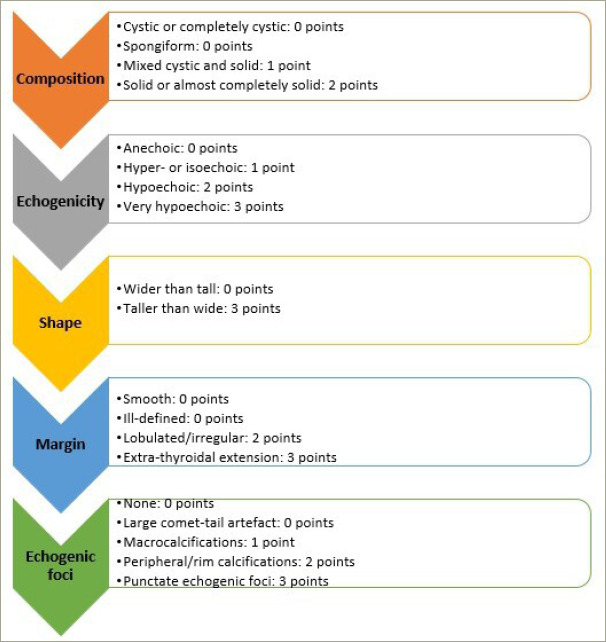
Scoring in the American College of Radiology thyroid imaging reporting and data system

ACR TI-RADS scoring is determined based on five distinct categories of US findings: composition, shape, echogenicity, margin and echogenic foci.^26^ Scoring is based on TI-RADS levels and follow-up US, and points are designated to each US feature, with higher values indicating a greater probability of suspicion. *Figure 1* displays these characteristics organized according to the five lexicon categories.^26^ The total score of a nodule is applied to allocate its TR category. A thyroid nodule with a total score of 0, 2, 3, 4–6 and 7 and more is categorized as TI-RADS 1, 2, 3, 4 and 5, respectively.^26^

### Chinese guidelines for thyroid imaging reporting and data system

Chinese TI-RADS (C-TIRADS) provides a validated method for assessing thyroid nodule malignancy risk .^40^ Developed in 2020 by the Superficial Organ and Vascular Ultrasound Group of the Society of Ultrasound in Medicine of the Chinese Medical Association, C-TIRADS is an updated version of TI-RADS.^41^ Unlike ACR TI-RADS, C-TIRADS focuses on six key US features that strongly indicate malignancy or benignity.^42^ These features include shape (orientation), composition (architecture), echogenicity, margin and echogenic foci. C-TIRADS uses a scoring scheme based on sonographic features, assigning one point for higher malignancy risk and deducting one point for benign features.^43^ The details of C-TIRADS categories and risk of malignancy are indicated in *Table 4*.^43^

Positive features of C-TIRADS include vertical orientation (+1), solid composition (+1), marked hypoechogenicity (+1), microcalcifications (+1), and ill-defined, irregular, lobulated or extrathyroidal extension margin (+1), and negative features are hyperechoic foci with a comet-tail artefact (-1). If there is more than one hyperechoic pattern in a nodule, only the highest score is entered.^44^

### Revised thyroid imaging reporting and data system

**Figure 2:**
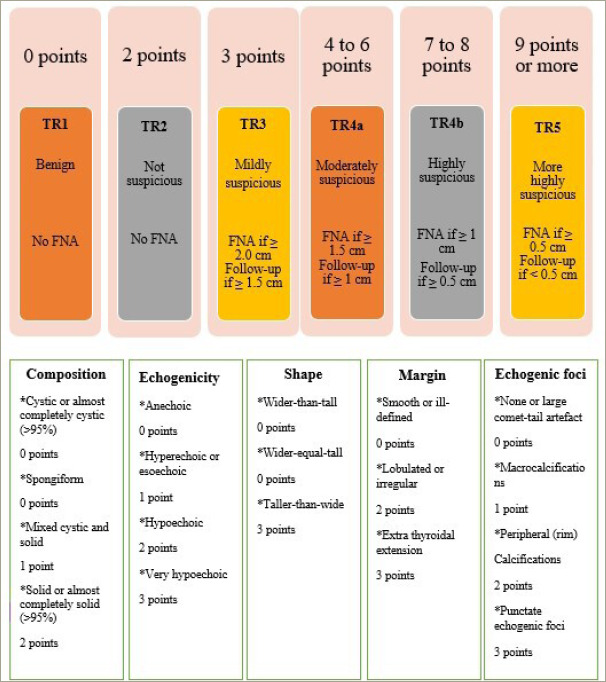
Six categories of the revised thyroid imaging reporting and data system, thyroid imaging reporting and data system levels and criteria for fine-needle aspiration or follow-up ultrasound^24^

Liang et al. introduced a pragmatic risk-stratification system, known as revised TI-RADS (R-TIRADS), which uses five US features, such as composition, echogenicity, shape, margin and echogenic foci, to categorize the malignancy risk associated with thyroid nodules effectively (Figure 2).^24^ The R-TIRADS imitates the ACR TI-RADS. They hypothesized that the R-TIRADS would improve the diagnostic sensitivity of thyroid nodules.^24^

**Table 2:** European thyroid imaging reporting and data system categories^21,34^

EU-TIRADS categories	Definition	US features	Risk of malignancy (%)
1	Normal	No nodule	NA
2	Benign	Pure cyst, entirely spongiform	0
3	Mildly suspicious	Ovoid, smooth isoechoic/hyperechoic	2–4
4	Moderately suspicious	Ovoid, smooth and mildly hypoechoic	6–17
5	Highly suspicious	One of the following features: Irregular shape Irregular margins Microcalcifications Marked hypoechogenicity (and solid)	26–87

*EU-TIRADS = European thyroid imaging reporting and data system; NA = not applicable; US = ultrasound.*

**Table 3:** American College of Radiology thyroid imaging reporting and data system categories^26,39^

ACR TI-RADS categories	Definition	Scoring	Risk of malignancy (%)	Recommendation
TR1	Benign	0 points	0.3	No FNA required
TR2	Not suspicious	2 points	1.5	No FNA required
TR3	Mildly suspicious	3 points	4.8	≥2.5 cm: FNA ≥1.5 cm: follow-up (for 1, 3 and 5 years)
TR4	Moderately suspicious	4–6 points	9.1	≥1.5 cm: FNA ≥1.0 cm: follow-up (for 1, 2, 3 and 5 years)
TR5	Highly suspicious	≥7 points	35	≥1.0 cm: FNA ≥0.5 cm: follow-up Annual follow-up for up to 5 years

*ACR = American College of Radiology; FNA = fine-needle aspiration; TR = thyroid imaging reporting and data system category.*

### Artificial intelligence thyroid imaging reporting and data system

Artificial intelligence TI-RADS (AI-TIRADS) uses artificial intelligence (AI) to optimize the TI-RADS. Wildman-Tobriner et al. posited that the AI-optimized TI-RADS is a data-driven model for the purpose of risk stratification of thyroid nodules.^45^ This model not only validates ACR TI-RADS, but also proposes modifications to it that may enhance its performance and applicability.

*The score is designated from each of these five categories: composition, echogenicity, shape, margin and echogenic foci. If there are several nodules, only the four highest-scoring nodules (not inevitably the largest) should be scored, reported and followed up. Mainly cystic or spongiform nodules are essentially benign. If these features are present, no additional points will be included (automatically as thyroid imaging reporting and data system category)*

**Table 4:** Chinese thyroid imaging reporting and data system categories^43^

C-TIRADS categories	Definition	Scoring	Risk of malignancy (%)
1	A nodule-free thyroid	NA	0
2	Benign	-1	0
3	Probably benign	0	<2
4A	Low suspicion	1	2–10
4B	Moderate suspicion	2	10–50
4C	High suspicion	3 or 4	50–90
5	Highly suggestive of malignancy	5	>90
6	A nodule with a malignancy	NA	NA

*C-TIRADS = Chinese thyroid imaging reporting and data system; NA = not applicable.*

*FNA = fine needle aspiration; TR = thyroid imaging reporting and data system category.*

## Similarities and differences between risk-stratification systems

Şahin et al. assessed the effectiveness of ATA and TI-RADS classifications to identify malignancy.^46^ They found that the sensitivity and specificity of the ATA risk classification were 80% and 96.3%, whereas the sensitivity and specificity of the TI-RADS classification were 76% and 97.5%. Accordingly, ATA and TI-RADS have high specificity and sensitivity. Moreover, they found that the best category for identifying malignant and benign nodules was TR5 for TI-RADS and the high-suspicion category for ATA.^46^

Yoon et al. conducted a study comparing K-TIRADS, ACR TI-RADS and EU-TIRADS.^25^ They also assessed the effectiveness of sonographic FNA criteria in detecting malignant thyroid nodules. While the three systems had similarities in most US lexicons and categorization systems, there were notable differences in how they classified thyroid nodules. The diagnostic performance of FNA size criteria varied significantly between these systems, primarily due to differences in FNA size criteria and partly due to diverse risk categorizations of nodules. Understanding the similarities and differences of these systems is important for improving international standardization.^25^

Approximately 2% of [^18^F]fluorodeoxyglucose PET/CT scans show focal thyroid incidentaloma (TI), with about one-third being cancerous.^47^ However, there is a lack of evidence on the best management approach for TI. Current guidelines suggest using FNA. In a study by Trimboli et al., the effectiveness of ACR TI-RADS, EU-TIRADS and K-TIRADS in determining the need for FNA in TIs was evaluated.^48^ The study found that all TI-RADSs successfully categorized the risk of cancer in focal TI. EU-TIRADS and K-TIRADS performed well in indicating the need for FNA. Given the high likelihood of cancer in this patient population, it is recommended to prioritize TI-RADS systems that are more likely to recommend FNA.^48^

The EU-TIRADS and ACR TI-RADS scores were implemented to guide clinicians in performing FNA. However, their effectiveness has not been evaluated or compared in patients undergoing surgery. Magri et al. conducted a study to assess the accuracy of these scores in real-l ife patients who underwent thyroidectomy.^28^ The study aimed to identify any cases of missed thyroid cancer due to these scores. The findings showed that both ACR TI-RADS and EU-TIRADS accurately predict thyroid cancer when histology is used as a reference standard. However, clinical judgement is still needed to determine the need for FNA.^28^

Watkins et al. compared the diagnostic capabilities of the British Thyroid Association (BTA), ACR TI-RADS and AI-TIRADS in detecting the malignancy in thyroid nodules.^49^ The study aimed to assess unnecessary FNA rates. Results showed that all systems had similar diagnostic performance, achieving a sensitivity of over 93%. Malignant nodules were classified as US grade 4–5, while benign nodules have grade 1–2. ACR TI-RADS and AI-TIRADS significantly reduced recommended FNA frequency for non-cancerous nodules compared with BTA.^49^

The US-based diagnosis of thyroid nodules has been extensively studied, and the ACR TI-RADS reporting system consistently shows superior accuracy compared with other systems. It effectively reduces the need for biopsies of benign nodules. In a recent study by Hoang et al., the frequency of recommended FNA for low-risk adult patients undergoing sonographic evaluation was examined.^50^ ACR TI-RADS suggests 25–50% fewer biopsies compared with ATA, EU-TIRADS and K-TIRADS due to differences in size thresholds and risk-l evel criteria.

## Diagnostic value of thyroid imaging reporting and data system

As the incidence of thyroid nodules is increasing around the globe, accurate interpretation of thyroid nodule features is an extremely important component of any classification system.^51^

Thyroid biopsy methods are different among practitioners. There is contradictory use of resources, such as on-site cytopathologists who ascertain if biopsy samples are sufficient for diagnosis. Providers who perform diagnostic procedures can compare quality measures, such as diagnostic output, false-negative rate and false-positive rate. This comparison helps ensure that optimal procedural methods are followed and unnecessary repeat biopsies are avoided.^52^

Schenke et al. recommended including scintigraphy in the TI-RADS model to identify hyperfunctioning thyroid nodules and reduce unnecessary diagnostic procedures.^53^ This improves the positive predictive value (PPV) of a high TI-RADS class. However, scintigraphy carries a potential risk of inducing hyperthyroidism. Their findings about the importance of scintigraphy are consistent with publications by other researchers.

Chandramohan et al. found that TI-RADS is a practical method for assessing thyroid nodules with high PPV and agreement among observers.^38^ They conducted a prospective study to evaluate the PPV and interobserver agreement of TI-RADS. They performed US on patients with thyroid nodules of >1 cm. US features and the TI-RADS category were compared with cytology and surgical histopathology. The determination of the PPV and likelihood ratio for malignancy, based on ultrasound features of thyroid nodules and final assessment categories, was conducted using data from the assessments of all readers combined. Moreover, interobserver agreement was calculated using linear weighted kappa. As a result, they indicated that TI-RADS is a simple and practical technique for evaluating thyroid nodules, which can be used in practice.^38^

Wei et al. evaluated the overall diagnostic accuracy of TI-RADS categorization in the differential diagnosis of patients with thyroid nodules.^51^ Using meta-analysis techniques, specificity, pooled sensitivity, positive likelihood ratio, negative likelihood ratio, diagnostic odds ratio and summary receiver operating characteristic curves were obtained. Consequently, the TI-RADS classification proves to be a reliable diagnostic approach for distinguishing thyroid nodules.

Ahn et al. conducted a retrospective study on 432 thyroid nodules to assess the false-negative rate in US-guided FNA.^54^ The study found an overall false-negative rate of 3.2%. The study also found that as the K-TIRADS score increased, the false-negative rate tended to be higher. In nodules with low or high suspicion (K-TIRADS 3 and 5), there was no significant difference in the false-negative rate based on nodule size. However, in nodules with intermediate suspicion (K-TIRADS 4), larger nodules had a higher false-negative rate. Therefore, the impact of nodule size on the false-negative rate varied depending on the US pattern.

## Limitations

There are certain limitations associated with this study, including the presence of both similarities and discrepancies in terminology and standards used to describe and define the characteristics of thyroid nodules in the US feature, as different researchers develop their classification systems.

## Challenges and problems

Applying new guidelines for the evaluation of a specific condition or organ system often poses some challenges and problems.^24,26,33,34,45,46^ Setting up TI-RADS can present issues associated with measurement accuracy, imaging and workflow, structured reporting, interpretation, interobserver variability, performance in detecting non-PTC and implementation and quality improvement.^26,52^

### Measurement accuracy and reproducibility

Thyroid nodules must be sized accurately, as the maximum dimension specifies whether a given lesion needs to be biopsied or followed. While it is inevitable to have some differences in observations among observers due to the variability of the variable, the use of a consistent approach improves the accuracy and reproducibility of measurements.^26^ Nodules should be measured in three dimensions: (1) the largest dimension on an axial image, (2) the largest dimension perpendicular to a previous measurement on the same image and (3) the greatest longitudinal dimension on a sagittal image.^55^

### Imaging and workflow

The issues with TI-RADS stem from education, workflow and interpretation. Educating and training sonographers is crucial for implementing TI-RADS worldwide. Consequently, implementing a standardized sonographer protocol becomes an essential prerequisite for ensuring uniformity in the quality of service provided.^30^ Thyroid nodules measuring less than 5 mm should not be given much attention. For nodules larger than 5 mm, it is necessary to obtain still images and cine clips in both transverse and sagittal planes, focusing on the relevant US characteristics. The size of each nodule is determined by measuring its anterior–posterior, transverse and craniocaudal dimensions.^52^

Furthermore, developing a worksheet or annotation system to help the sonographer in documenting the features of nodules and other data is essential. A thyroid gland illustration could be included to indicate the location of a nodule, with adjacent spaces where measurements and TI-RADS-specific features are recorded. If the sonogram is conducted for follow-up purposes, it is advisable for the sonographer to thoroughly examine previous images and reports in order to identify any mentioned and measured nodules, whenever feasible.^30^

### Structured reporting

Generating a comprehensive and well-organized report for a thyroid examination poses a significant challenge for radiologists, especially in cases where there are multiple nodules. Accordingly, the sonographer should recognize and catalogue a maximum of four nodules that are ranked by point and measure them.^52^ Structured reporting can increase report homogeneity and language uniformity, allowing a standardized approach and recommendations, causing better communication between radiologists and clinicians and eliminating radiologist individuality.^56^ Three tiers of structured reporting exist. The initial tier involves the usage of headings, such as indications, comparison, findings and impression. The second tier incorporates organ systems as subheadings, also known as itemized reporting. The third tier incorporates standardized terminology and language. TI-RADS reports are particularly suitable for the third tier, as they provide standardized terms to describe thyroid nodules.^56,57^

### Interpretation and interobserver variability

A precise understanding of the characteristics of thyroid nodules is an essential component of any classification system. While the TI-RADS parameters only permit the indexing of the four most concerning nodules, evaluating a thyroid gland with numerous nodules can pose a significant challenge.^58^

Tappouni et al. introduced an algorithmic approach that can aid in categorizing nodules for indexing purposes.^52^ They demonstrated that spongiform and cystic nodules, based on their composition, are benign and do not require any intervention. As a result, these characteristics would be given the least priority when reporting. If the nodule is not spongiform or cystic, then the existence and appearance of peripheral calcifications are evaluated, as these are suspicious features. All features of the echogenic foci options are incorporated and contributed to the overall score.

The definition of features is of significant importance, particularly with regard to the inclusion of a lexicon in certain systems. It should be noted that, for certain features, there may exist multiple definitions, such as in the case of taller-than-wide shapes. To ensure the appropriate implementation of sonographic risk-stratification systems, it is crucial to define the independent risk characteristics that form the basis of each system.^59^

Grani et al. conducted a study demonstrating that grey-scale sonographic features are independently linked to malignancy and compared various definitions.^59^ The key suspicious features of thyroid nodules were identified by the authors, who also offered a simplified definition for certain controversial aspects. Additionally, they noted that the malignancy rate increases in proportion to the number of features present.

Thyroid nodules with a taller-than-wide shape are often identified by an anteroposterior/transverse diameter (AP/T) ratio greater than 1. However, there is a variation in the assessment of the AP/T diameter, which could lead to overreporting. Grani et al. proposed a new ratio of ≥1.2 to improve the reliability of the taller-than-wide definition.^60^ They evaluated the diagnostic performance and found that redefining nodules with an AP/T ratio of ≥1.2 enhances the specificity for malignancy. By incorporating this definition into risk-stratification systems, the specificity will increase, reducing the number of suggested biopsies without compromising the overall diagnostic performance significantly.

In another investigation conducted by Li et al., the diagnostic efficacy of a novel ultrasonographic technique in assessing thyroid nodules with a taller-than-wide configuration was examined.^61^ The usage of their innovative ultrasonographic method to measure a taller-than-wide shape demonstrated exceptional accuracy in predicting the presence of thyroid malignancy.

### The performance in detecting non-papillary thyroid cancer

Trimboli et al. conducted a study to determine whether risk-stratification systems have been adequately investigated in all types of thyroid malignancies.^62^ The researchers conducted a thorough investigation by reviewing the studies that categorized thyroid nodules using five common US risk-stratification systems. These studies also included information on the histological diagnosis of cancerous lesions. Additionally, a meta-analysis was performed to determine the overall prevalence of cancer and the relative prevalence of different types of thyroid cancers, such as PTC, follicular thyroid cancer (FTC) and medullary thyroid cancer (MTC).

The study found that most confirmed cancers were PTCs, indicating that US classifications are reliable for diagnosing PTCs. However, to improve the detection of other types of thyroid cancers, such as FTCs and MTCs, the patterns and thresholds for FNA should be refined or US should be combined with other technologies. Furthermore, the results raise the question whether clinicians are focusing on detecting PTCs while neglecting the most aggressive thyroid cancers. Therefore, further studies are recommended to investigate this issue.^62^

The most challenging indeterminate nodules are those classified as atypia of undetermined significance/follicular lesion of undetermined significance (AUS/FLUS) and follicular or oncocytic (Hürthle cell) neoplasm/suspicious for a follicular or oncocytic (Hürthle cell) neoplasm (FN/SFN) according to The Bethesda System for Reporting Thyroid Cytopathology (TBSRTC) (Bethesda Category III and IV, respectively).^63^ The 2017 edition of TBSRTC revised the predicted probability of malignancy for indeterminate nodules, estimating it to be 10–30% for AUS/FLUS and 25–40% for FN/SFN when considering the non-i nvasive follicular thyroid neoplasm with papillary-l ike nuclear features as a malignant tumour. While it is recommended to follow up with patients and repeat FNA for AUS/FLUS nodules, FN/SFN nodules pose a more significant clinical challenge to address.^64^

Marina et al. revealed that the amalgamation of US risk-stratification systems and molecular testing (e.g. v-raf murine sarcoma viral oncogene homolog B [BRAF] and rat sarcoma [RAS] mutation analyses) enhances the evaluation of malignancy risk in TBSRTC IV thyroid nodules in contrast to singular assessments.^64^

### Implementation and quality improvement

There are two national quality measures for thyroid nodules: Merit-based Incentive Payment System (MIPS) measures 406 and 265.^65^ Measure 406 looks at incidental nodules found during CT and MRI scans, while measure 265 ensures thorough review and communication of biopsy results. Quality measures can be assessed in the fields of efficiency, diagnostic accuracy, appropriateness and patient-centred care.^66^

## Future perspectives and strengths

TI-RADS has some strengths in categorizing the risk of malignancy, making predictions about malignant thyroid nodules and guiding clinical decision-making based on additional information, as determined by a healthcare professional. TI-RADS should be updated as new information becomes available. Hoang et al. found that because thyroid cancer grows slowly and active surveillance has positive results, it is important to consider a larger size threshold for recommending FNA for TR3–TR5 nodules.^67^ One adjustment could be considering the nodule’s location in the evaluation. A study found a higher chance of malignancy in nodules located in the isthmus.^68^ Another change could be revising the point system for certain characteristics. For instance, a study proposed assigning less than three points to punctate echogenic foci when they appear in nodules with both cystic and solid components.^69^

Incorporating US techniques, such as elastography and contrast-enhanced US, into future ACR TI-RADS updates is recommended. Elastography is a reliable way to assess tissue stiffness in malignant nodules and can help identify abnormal organ stiffness.^70–73^ Combining elastography with B-mode US features has been shown to improve diagnosis accuracy, increasing both sensitivity and specificity.^74,75^

Moreover, Tappouni et al. revealed that other structured reporting systems, such as BI-RADS and liver imaging reporting and data system (LI-RADS), contain categories that can be implemented into TI-RADS, for instance, a thyroid nodule that is recognized to be malignant but has not been or potentially will not be treated.^52^ This would be similar to the BI-RADS 6 category and may be particularly important when patient care is transferred to another practice. Therefore, a repeat and possibly discordant assessment can be avoided.

On the contrary, patients with a history of thyroidectomy or ablation who present with abnormal imaging findings should not receive an evaluation out of context. These patients may take advantage of a posttreatment category as described in LI-RADS (e.g. TR viable or TR non-viable).^52^ Additionally, individuals with uncertain nodules and negative or inconclusive FNA results should have a follow-up biopsy or be referred to a surgeon for possible thyroidectomy. For instance, a TR5 lesion with negative FNA findings should experience a repeat biopsy, as a sampling error may have happened. As a result, a particular suggestion for a repeat biopsy or surgery referral may assist in such conditions.^52^

Furthermore, Medas et al. suggest that the decrease in surgeries for uncertain thyroid nodules during the coronavirus disease 2019 (COVID-19) pandemic may have led to an increase in aggressive thyroid tumours.^76^ However, other theories, such as the selection of patients with aggressive malignancies, should also be considered. They recommend avoiding postponing surgery for uncertain thyroid nodules in future pandemics.

AI has revolutionized the assessment of thyroid nodules, being used effectively in US, CT scans and MRI scans.^77^ In certain cases, AI systems can even accurately predict the immunohistochemistry of thyroid nodules by evaluating segmented image data sets. Furthermore, the integration of computer-assisted diagnosis (CAD) into daily clinical practice does not significantly disrupt the workflow, as it only increases the examination time by approximately 2-3 minutes.^78^

However, AI systems still have a long way to go before they can fully replace experienced radiologists in terms of improving accuracy and reducing time consumption. Larger studies that meet uniformity criteria are necessary to further evaluate the diagnostic performance of these systems. Nonetheless, current CAD systems provide valuable support to radiologists in the assessment of thyroid nodules and contribute to an overall increase in the accuracy of routine thyroid US.^79^

A universal risk-stratification system is urgently needed to assist both clinicians and patients in understanding US reports and making informed decisions about nodules that require further evaluation, such as a biopsy. At present, the International Thyroid Nodule Ultrasound Working Group is leading an effort to create an international risk-stratification system called I-TIRADS. This system will incorporate the best risk-stratification systems currently available.^80^

A universal risk-stratification system and standardized terminology are needed to simplify thyroid nodule evaluation and reduce unnecessary biopsies while identifying significant malignancies. I-TIRADS is a significant step towards this goal; however, further validation is needed through population studies before implementation.^40^

## Conclusions

The prevalence of thyroid nodules is on the rise globally, posing a significant threat to human health. Therefore, it is imperative to enhance the detection of aggressive thyroid cancers. Recent advancements in treatment guidelines aim to minimize unnecessary treatment. Consequently, it is crucial to establish an accurate risk-stratification system to avoid overdiagnosis, excessive treatment and unnecessary imaging and biopsies. The TI-RADS classification serves as a reliable diagnostic tool for distinguishing between benign and malignant thyroid nodules. This system provides a structured reporting framework and consistent nodule classification, enabling appropriate management recommendations. However, there are certain challenges in implementing this system. Currently, AI is transforming operations by reducing human involvement and using computers to imitate intelligent actions. Deep learning, a cutting-edge technology, has made significant advancements by enabling models to learn from data without explicit instructions. With this remarkable technology, AI is rapidly advancing and finding applications in various domains. AI-TIRADS has the potential to enhance the accuracy of thyroid cancer diagnosis, differentiate between benign and malignant thyroid conditions and promote interobserver agreement, particularly those who are less experienced in this field. As a result, the TI-RADS holds potential for various research opportunities and future modifications. It is essential to conduct unified TI-RADS classification criteria and high-quality prospective studies to improve the diagnosis of thyroid nodules.

## References

[1] Alexander EK, Cibas ES (2022). Diagnosis of thyroid nodules.. Lancet Diabetes Endocrinol..

[2] Ward EM, Sherman RL, Henley SJ (2019). Annual report to the nation on the status of cancer, featuring cancer in men and women age 20–49 years.. J Natl Cancer Inst..

[3] Wong R, Farrell SG, Grossmann M (2018). Thyroid nodules: Diagnosis and management.. Med J Aust..

[4] Anwar K, Mohammad AY, Khan S (2023). The sensitivity of TIRADS scoring on ultrasonography in the management of thyroid nodules.. Pak J Med Sci..

[5] Han M, Ha EJ, Park JH (2021). Computer-aided diagnostic system for thyroid nodules on ultrasonography: Diagnostic performance based on the thyroid imaging reporting and data system classification and dichotomous outcomes.. AJNR Am J Neuroradiol..

[6] Fisher SB, Perrier ND (2018). The incidental thyroid nodule.. CA Cancer J Clin..

[7] Kitahara CM, Schneider AB (2022). Epidemiology of thyroid cancer.. Cancer Epidemiol Biomarkers Prev..

[8] Megwalu UC, Moon PK (2022). Thyroid cancer incidence and mortality trends in the United States: 2000–2018.. Thyroid..

[9] Furuya-Kanamori L, Bell KJL, Clark J (2016). Prevalence of differentiated thyroid cancer in autopsy studies over six decades: A meta-analysis.. J Clin Oncol..

[10] Ottino A, Pianzola HM, Castelletto RH (1989). Occult papillary thyroid carcinoma at autopsy in La Plata, Argentina.. Cancer..

[11] Sampson RJ, Oka H, Key CR (1970). Metastases from occult thyroid carcinoma. An autopsy study from Hiroshima and Nagasaki, Japan.. Cancer..

[12] LeClair K, Bell KJL, Furuya-Kanamori L (2021). Evaluation of gender inequity in thyroid cancer diagnosis: Differences by sex in US thyroid cancer incidence compared with a meta-analysis of subclinical thyroid cancer rates at autopsy.. JAMA Intern Med..

[13] Hoang VT, Trinh CT (2020). A review of the pathology, diagnosis and management of colloid goitre.. Eur Endocrinol..

[14] Sanabria A, Kowalski LP, Shah JP (2018). Growing incidence of thyroid carcinoma in recent years: Factors underlying overdiagnosis.. Head & Neck..

[15] Grodski S, Brown T, Sidhu S (2008). Increasing incidence of thyroid cancer is due to increased pathologic detection.. Surgery..

[16] Gharib H, Papini E, Paschke R (2010). American Association of Clinical Endocrinologists, Associazione Medici Endocrinologi, and European Thyroid Association medical guidelines for clinical practice for the diagnosis and management of thyroid nodules: Executive summary of recommendations.. Endocr Pract..

[17] Luster M, Aktolun C, Amendoeira I (2019). European perspective on 2015 American Thyroid Association management guidelines for adult patients with thyroid nodules and differentiated thyroid cancer: Proceedings of an interactive international symposium.. Thyroid..

[18] Powers AE, Marcadis AR, Lee M (2019). Changes in trends in thyroid cancer incidence in the United States.. JAMA..

[19] Gharib H, Papini E, Paschke R (2010). American Association of Clinical Endocrinologists, Associazione Medici Endocrinologi, and European Thyroid Association medical guidelines for clinical practice for the diagnosis and management of thyroid nodules: Executive summary of recommendations.. J Endocrinol Invest..

[20] Nabhan F, Dedhia PH, Ringel MD (2021). Thyroid cancer, recent advances in diagnosis and therapy.. Int J Cancer..

[21] Kwak JY, Han KH, Yoon JH (2011). Thyroid imaging reporting and data system for US features of nodules: A step in establishing better stratification of cancer risk.. Radiology..

[22] Kim DW, Park JS, In HS (2012). Ultrasound-based diagnostic classification for solid and partially cystic thyroid nodules.. AJNR Am J Neuroradiol..

[23] Horvath E, Majlis S, Rossi R (2009). An ultrasonogram reporting system for thyroid nodules stratifying cancer risk for clinical management.. J Clin Endocrinol Metab..

[24] Liang F, Li X, Ji Q (2023). Revised thyroid imaging reporting and data system (TIRADS): Imitating the American College of Radiology TIRADS, a single-center retrospective study.. Quant Imaging Med Surg..

[25] Yoon SJ, Na DG, Gwon HY (2019). Similarities and differences between thyroid imaging reporting and data systems.. AJR Am J Roentgenol..

[26] Tessler FN, Middleton WD, Grant EG (2017). ACR thyroid imaging, reporting and data system (TI-RADS): White paper of the ACR TI-RADS committee.. J Am Coll Radiol..

[27] Grant EG, Tessler FN, Hoang JK (2015). Thyroid ultrasound reporting lexicon: White paper of the ACR thyroid imaging, reporting and data system (TIRADS) committee.. J Am Coll Radiol..

[28] Magri F, Chytiris S, Croce L (2020). Performance of the ACR TI-RADS and EU TI-RADS scoring systems in the diagnostic work-up of thyroid nodules in a real-l ife series using histology as reference standard.. Eur J Endocrinol..

[29] Haugen BR, Alexander EK, Bible KC (2016). American Thyroid Association management guidelines for adult patients with thyroid nodules and differentiated thyroid cancer: The American Thyroid Association guidelines task force on thyroid nodules and differentiated thyroid cancer.. Thyroid..

[30] Rossi ED, Pantanowitz L, Raffaelli M, Fadda G (2021). Overview of the ultrasound classification systems in the field of thyroid cytology.. Cancers (Basel)..

[31] Kwak JY, Jung I, Baek JH (2013). Image reporting and characterization system for ultrasound features of thyroid nodules: Multicentric Korean retrospective study.. Korean J Radiol..

[32] Zhang W-B, Xu W, Fu W-J (2021). Comparison of ACR TI-RADS, Kwak TI-RADS, ATA guidelines and KTA/KSThR guidelines in combination with SWE in the diagnosis of thyroid nodules.. Clin Hemorheol Microcirc..

[33] Shin JH, Baek JH, Chung J (2016). Ultrasonography diagnosis and imaging-based management of thyroid nodules: Revised Korean Society of Thyroid Radiology consensus statement and recommendations.. Korean J Radiol..

[34] Russ G, Bonnema SJ, Erdogan MF (2017). European Thyroid Association guidelines for ultrasound malignancy risk stratification of thyroid nodules in adults: The EU-TIRADS.. Eur Thyroid J..

[35] Na DG, Baek JH, Sung JY (2016). Thyroid imaging reporting and data system risk stratification of thyroid nodules: Categorization based on solidity and echogenicity.. Thyroid..

[36] Horvath E, Silva CF, Majlis S (2017). Prospective validation of the ultrasound based TIRADS (thyroid imaging reporting and data system) classification: Results in surgically resected thyroid nodules.. Eur Radiol..

[37] Hoang JK, Langer JE, Middleton WD (2015). Managing incidental thyroid nodules detected on imaging: White paper of the ACR Incidental Thyroid Findings Committee.. J Am Coll Radiol..

[38] Chandramohan A, Khurana A, Pushpa BT (2016). Is TIRADS a practical and accurate system for use in daily clinical practice. Indian J Radiol Imaging..

[39] Middleton WD, Teefey SA, Reading CC (2017). Multiinstitutional analysis of thyroid nodule risk stratification using the American College of Radiology Thyroid Imaging Reporting and Data System.. AJR Am J Roentgenol..

[40] Majety P (2023). Thyroid nodules: Need for a universal risk stratification system.. Front Endocrinol (Lausanne)..

[41] Zhou J, Yin L, Wei X Superficial Organ and Vascular Ultrasound Group of the Society of Ultrasound in Medicine of the Chinese Medical Association; Chinese Artificial Intelligence Alliance for Thyroid and Breast Ultrasound 2020 Chinese guidelines for ultrasound malignancy risk stratification of thyroid nodules: the C-TIRADS.. Endocrine.

[42] Chen Q, Lin M, Wu S (2022). K-TIRADS and ACR-TIRADS in stratifying the malignancy risk of thyroid nodules.. Front Endocrinol..

[43] Hu Y, Xu S, Zhan W (2022). Diagnostic performance of C-TIRADS in malignancy risk stratification of thyroid nodules: A systematic review and meta-analysis.. Front Endocrinol..

[44] Zhou J, Yin L, Wei X (2020). Chinese guidelines for ultrasound malignancy risk stratification of thyroid nodules: The C-TIRADS.. Endocrine..

[45] Wildman-Tobriner B, Buda M, Hoang JK (2019). Using artificial intelligence to revise ACR TI-RADS risk stratification of thyroid nodules: Diagnostic accuracy and utility.. Radiology..

[46] Şahin M, Oguz A, Tuzun D (2021). Effectiveness of TI-RADS and ATA classifications for predicting malignancy of thyroid nodules.. Adv Clin Exp Med..

[47] Scappaticcio L, Piccardo A, Treglia G (2021). The dilemma of 18F-FDG PET/CT thyroid Incidentaloma: What we should expect from FNA. A systematic review and meta-analysis.. Endocrine..

[48] Trimboli P, Knappe L, Treglia G (2020). FNA indication according to ACR-TIRADS, EU-TIRADS and K-TIRADS in thyroid incidentalomas at 18 F-FDF PET/CT.. J Endocrinol Invest..

[49] Watkins L, O’Neill G, Young D, McArthur C (2021). Comparison of British Thyroid Association, American College of Radiology TIRADS and artificial intelligence TIRADS with histological correlation: Diagnostic performance for predicting thyroid malignancy and unnecessary fine needle aspiration rate.. Br J Radiol..

[50] Hoang JK, Middleton WD, Langer JE (2021). Comparison of thyroid risk categorization systems and fine-needle aspiration recommendations in a multi-i nstitutional thyroid ultrasound registry.. J Am Coll Radiol..

[51] Wei X, Li Y, Zhang S, Gao M (2014). Thyroid imaging reporting and data system (TI-RADS) in the diagnostic value of thyroid nodules: A systematic review.. Tumor Biol..

[52] Tappouni RR, Itri JN, McQueen TS (2019). ACR TI-RADS: Pitfalls, solutions, and future directions.. Radiographics..

[53] Schenke S, Seifert P, Zimny M (2019). Risk stratification of thyroid nodules using the thyroid imaging reporting and data system (TIRADS): The omission of thyroid scintigraphy increases the rate of falsely suspected lesions.. J Nucl Med..

[54] Ahn HS, Na DG, Baek JH (2019). False negative rate of fine-needle aspiration in thyroid nodules: Impact of nodule size and ultrasound pattern.. Head Neck..

[55] Choi YJ, Baek JH, Hong MJ, Lee JH (2015). Inter-observer variation in ultrasound measurement of the volume and diameter of thyroid nodules.. Korean J Radiol..

[56] Granata V, De Muzio F, Cutolo C (2022). Structured reporting in radiological settings: Pitfalls and perspectives.. J Pers Med..

[57] Ma J, Wu F, Jiang T (2017). Ultrasound image-based thyroid nodule automatic segmentation using convolutional neural networks.. Int J Comput Assist Radiol Surg..

[58] Ma J, Wu F, Jiang T (2017). Ultrasound image-based thyroid nodule automatic segmentation using convolutional neural networks.. Int J Comput Assist Radiol Surg..

[59] Grani G, Del Gatto V, Cantisani V (2023). A reappraisal of suspicious sonographic features of thyroid nodules: Shape is not an independent predictor of malignancy.. J Clin Endocrinol Metab..

[60] Grani G, Lamartina L, Ramundo V (2020). Taller-than-wide shape: A new definition improves the specificity of TIRADS systems.. Eur Thyroid J..

[61] Li C, Xin X, Wang X (2023). The diagnostic value of a new ultrasonographic method for the measurement of a taller-than-wide shape of benign and malignant thyroid nodules.. Endocrine..

[62] Trimboli P, Castellana M, Piccardo A (2021). The ultrasound risk stratification systems for thyroid nodule have been evaluated against papillary carcinoma. A meta-analysis.. Rev Endocr Metab Disord..

[63] Rai K, Park J, Gokhale S (2023). Diagnostic accuracy of the Bethesda System for Reporting Thyroid Cytopathology (TBSRTC): An institution experience.. Int J Endocrinol..

[64] Marina M, Zatelli MC, Goldoni M (2021). Combination of ultrasound and molecular testing in malignancy risk estimate of Bethesda category IV thyroid nodules: Results from a single-institution prospective study.. J Endocrinol Invest..

[65] Noor M, Bivins E, Manchec B (2021). “Current Interventional Radiology-related Benchmarked clinical quality measures are less likely to be “capped” than diagnostic Radiology clinical quality measure.”.. J Vasc Interv Radiol..

[66] Chan AJ, Sarrazin J, Halperin IJ (2022). Quality improvement initiative to standardise thyroid ultrasound reports and reduce unnecessary fine-needle aspiration biopsies of thyroid nodules.. BMJ Open Qual..

[67] Hoang JK, Middleton WD, Tessler FN (2021). Update on ACR TI-RADS: Successes, challenges, and future directions, from the AJR special series on radiology reporting and data systems.. AJR Am J Roentgenol..

[68] Jasim S, Baranski TJ, Teefey SA, Middleton WD (2020). Investigating the effect of thyroid nodule location on the risk of thyroid cancer.. Thyroid..

[69] Teefey SA, Middleton WD, Reading CC (2021). Effect of decreasing the ACR TI-RADS point assignment for punctate echogenic foci when they occur in mixed solid and cystic thyroid nodules.. AJR Am J Roentgenol..

[70] Zhao C-K, Xu H-X (2019). Ultrasound elastography of the thyroid: Principles and current status.. Ultrasonography..

[71] Grgurevic I, Bokun T, Salkic NN (2018). Liver elastography malignancy prediction score for noninvasive characterization of focal liver lesions.. Liver Int..

[72] Quarato CMI, Venuti M, Dimitri L (2022). Transthoracic ultrasound shear wave elastography for the study of subpleural lung lesions.. Ultrasonography..

[73] Yang H, Xu Y, Zhao Y (2020). The role of tissue elasticity in the differential diagnosis of benign and malignant breast lesions using shear wave elastography.. BMC Cancer..

[74] Sigrist RMS, Liau J, Kaffas AE (2017). Ultrasound elastography: Review of techniques and clinical applications.. Theranostics..

[75] Du Y-R, Ji C-L, Wu Y, Gu X-G (2018). Combination of ultrasound elastography with TI-RADS in the diagnosis of small thyroid nodules (≤ 10 mm): A new method to increase the diagnostic performance.. Eur J Radiol..

[76] Medas F, Dobrinja C, Al-Suhaimi EA (2023). Effect of the COVID-19 pandemic on surgery for indeterminate thyroid nodules (THYCOVID): A retrospective, international, multicentre, cross-sectional study.. Lancet Diabetes Endocrinol..

[77] Chai YJ, Song J, Shaear M, Yi KH (2020). Artificial intelligence for thyroid Nodule ultrasound image analysis.. Ann Thyroid..

[78] Sorrenti S, Dolcetti V, Radzina M (2022). Artificial intelligence for thyroid nodule characterization: Where are we standing. Cancers (Basel)..

[79] Zhao W-J, Fu L-R, Huang Z-M (2019). Effectiveness evaluation of computer-aided diagnosis system for the diagnosis of thyroid nodules on ultrasound: A systematic review and meta-analysis.. Medicine (Baltimore)..

[80] Hoang JK, Asadollahi S, Durante C (2022). An international survey on utilization of five thyroid nodule risk stratification systems: A needs assessment with future implications.. Thyroid..

